# The Ether-Chloroform Controversy

**Published:** 1897-10

**Authors:** 


### THE ETHER-CHLOROFORM CONTROVERSY.

It is no exaggeration to say that no subject in medicine has enlisted the attention of more eminent investigators or consumed more printer's ink than the question of the relative advantages, disadvantages, and dangers of the two rival anesthetics, ether and chloroform. Surely the many thousands of surgical operations under both anesthetics which have been performed during the half century of the existence of anesthesia should have furnished an array of statistics competent to put the question empirically beyond controversy, while the physiological skill enlisted in its elucidation during the last twenty-five years would seem sufficient to have given it a final scientific settlement. Nevertheless the question is still open, and surgeons without prejudice still stand in doubt in certain cases as to whether this or that anesthetic should be employed.

Of course the extremists will shut their eyes to facts and continue to go each his own way; but the novice in surgery and the unprejudiced riper surgeon must still give the subject careful study and anxious thought. And indeed it does begin to look as if the question was about to have the scientific settlement so long looked for.

For decidedly to the point is the address on "The Causation of Chloroform Syncope," by Leonard Hill, M. B., Lecturer on Physiology at the London Hospital, delivered before the London Society of Anesthetists on February 18, 1897, and published in the *British Medical Journal* for April 17, 1897. In the opinion of the learned editor of the *Boston Medical and Surgical Journal* (from whose review of this paper we quote), Mr. Hill "makes some remarks upon this subject which, coming from England, where, as is well known, chloroform for many years completely displaced ether, and even now is the anesthetic most frequently used, are of especial interest."

Mr. Hill would seem to hint that the English have minimized the dangers, while perhaps overstating the advantages, of chloroform anesthesia; for it is a fact, attested by statistics accumulating during many years, that one death under ether occurs in about fifteen thousand administrations, "while chloroform causes one death in three thousand administrations."

"In a certain institution in Great Britain, in the course of a recent year, there were, out of some three or four thousand administrations, no fewer than twelve fatalities. This is no exceptional case. The deaths from chloroform are not recorded in the medical journals, for these reflect upon the reputation of the administrator and the institution in which they occur."

But, these points aside, the one great feature of the paper is that it claims to be a successful movement "toward the establishment of the true pathological causes of chloroform syncope, and the *controversion of one of the most pernicious and dangerous doctrines ever put before the medical profession*," to wit, that chloroform kills by paralyzing the respiratory center. In this the author flies in the face of the celebrated Hyderabad Commission, which, in putting fourth this doctriue, he claims was "supported by the wealth of the Hyderabad Government, furthered by the prejudiced enthusiasm of Surgeon-Lieutenant-Colonel Lawrie, and upheld by a series of experiments, many so careless that they could not for a moment be accepted by a trained physiologist." He might have added that the Commission so imposed upon the credulity of Dr. Lauder Brunton as to convert him to views which he had for many years ably disputed by theory and disproved by experiment. Thus "this doctrine that paralysis of the respiratory center causes chloroform syncope has been industriously spread abroad and instilled into the minds of the whole medical world."

In refutation of this primary dogma of the anesthetist's creed, Mr. Hill brings forward the following established facts:

1. Chloroform produces a fall of arterial pressure. This was proved by Snow, by the Glasgow Commission and others, long ago.

2. That the Hyderabad Commission, "while admitting this fact as incontrovertible, also found (by careless and inadequately planned experiments) that the respiratory center became paralyzed before the heart." This was disproved by MacWilliam, "who found, in several animals, sudden failure of the heart during primary anesthetization, while the respiration remained unaffected. This failure was found to be due to paralytic dilatation of the heart."

3. "Gaskel and Shore and Hare and Thornton found that the injection of chloroform into the jugular veins produced cardiac *followed* and not preceded by respiratory failure," a fact which the Hyderabad Commission failed to find, because they used pure instead of diluted chloroform, and because of badly planned and badly executed methods of experimentation." Gaskel and Shore found by careful and ingenious experimental methods that the heart is rapidly paralyzed by chloroform; the respiratory center is also paralyzed, but the vaso-motor center is excited to increased action."

In addition to this review of the work and refutation of the doctrine promulgated by the Hyderabad Commission the paper gives the details of considerable experimental work by the author, assisted by Harold Barnard, M. S., F. R. C. S., and C. Wall, B. A., of the London Hospital. In this work,

"change of position upon the circulation, with or without anesthesia, the effect of chloroform upon cardiac inhibition by electrical excitation of the vagus, the phenomena of fatal syncope at an early stage, syncope during prolonged anesthesia, and the treatment of syncope," are dealt with in a manner both skillful and scientific. His conclusions, which are of great practical interest to the surgeon, are as follows:

1. Cloroform produces a primary failure of the circulatory mechanism and a secondary failure of the respiratory center. The respiratory center fails to act not only because it is damaged by the drug, but because of the anemia of the spinal bulb produced by the fall of arterial tension. This is proved by the fact that the action of the respiratory center can be renewed by raising the arterial tension. The depth of anesthesia depends, as does the paralysis of the respiratory center, on the primary fall of the arterial tension.

2. Chloroform, more than any other known agent, rapidly abolishes the vascular mechanisms which compensate for the hydrostatic effect of gravity.

3. Chloroform abolishes these mechanisms by paralyzing the splanchnic vaso-motor tone, and by weakening the action of the respiratory pump. When these mechanisms are totally abolished, the circulation is impossible if the subject be in the feet-down position.

4. Chloroform also produces paralytic dilation of the heart. It acts directly like amyl nitrite on the musculature of the whole vascular system.

5. There are two forms of chloroform syncope: (*a*) During primary anesthetization. The patient struggles, holds his breath, raises the intrathoracic pressure, congests his venous system, lowers his arterial tension, and finally takes deep inspirations and surcharges his lungs with chloroform. In the first stage the left heart becomes impoverished; in the second stage it is suddenly filled with blood. This is drawn from the lungs, and is full of chloroform. The chloroform passes into the coronary arteries, and the heart is thrown into paralytic dilation. Respiration and the pulse either cease simultaneously, or the pulse before respiration. (*b*) During prolonged anesthetization this arises from gradually giving chloroform to too great an extent. The arterial pressure falls lower and lower, and secondarily, the respiration ceases because of the anemia of the spinal bulb. The heart is not in this case paralyzed by chloroform, because the drug is taken in gradually by the shallow respirations and distributed by the feeble circulation.

6. Artificial respiration and the assumption of the horizontal position, if applied in time, will always resuscitate a patient from the second form of syncope.

7. Artificial respiration, established with the patient in the horizontal posture, is also the treatment indicated in the first form of syncope; the heart should be rhythmically compressed by squeezing the thorax. If this does not quickly renew the pulse, the patient should be put into the vertical, feet-down posture. The dilated right heart is thereby completely and easily emptied of blood. Artificial respiration is maintained during this maneuver, and the patient is brought once more into the horizontal posture. By rhythmic compression of the chest an efficient circulation is maintained through the coronary arteries; by first emptying and then filling the heart a fresh supply of blood is brought into that organ. If this does not prove successful on the first trial it can be repeated.

8. Inversion, that is, placing the subject in the feet-up position, or compression of the abdomen will increase the paralytic dilation of the heart. In this kind of syncope both these forms of treatment are worse than useless.

9. In the condition of shock or emotional fear the compensatory mechanism for the effect of gravity is almost abolished, and chloroform may easily be the last straw to completely paralyze the circulation.

10. Vagus inhibition of the heart is of no importance as an agent in the production of chloroform syncope.

11. Ether is in every respect a far safer anesthetic than chloroform. According to Ringer's experiments on the heart, ether is fifty times less dangerous than chloroform.

12. With the practical conclusion of the Hyderabad Commission that the chloroform inhaler should be removed during the struggling of the patient or when the respiration is of irregular depth, I am in absolute agreement, but I consider their interpretation of their own experiments and tracings concerning the origin of chloroform syncope to be mistaken.

Not only the work of all physiologists, but also the tracings of the Commission, when rightly interpreted, prove that paralysis of the circulatory mechanism, and not of the respiratory center, is to be dreaded by the anesthetist.—*American Practitioner and News.*

